# VI-RADS score system - A primer for urologists

**DOI:** 10.1590/S1677-5538.IBJU.2021.0560

**Published:** 2022-02-02

**Authors:** Refky Nicola, Martina Pecoraro, Sara Lucciola, Rodolfo Borges dos Reis, Yoshifumi Narumi, Valeria Panebianco, Valdair Francisco Muglia

**Affiliations:** 1 Roswell Park Comprehensive Cancer Center Buffalo NY USA Roswell Park Comprehensive Cancer Center , Buffalo , NY , USA ;; 2 Department of Radiological Sciences, Oncology and Pathology Sapienza University of Rome Italy Department of Radiological Sciences, Oncology and Pathology , Sapienza University of Rome , Italy ;; 3 Departamento de Cirurgia, Divisão de Urologia Faculdade de Medicina de Ribeirão Preto USP Ribeirão Preto SP Brasil Departamento de Cirurgia, Divisão de Urologia - Faculdade de Medicina de Ribeirão Preto – USP , Ribeirão Preto , SP , Brasil ;; 4 Department of Health Care Kyoto Tachibana Kyoto Japan Department of Health Care , Kyoto Tachibana , Kyoto , Japan ;; 5 Departamento de Imagens Médicas, Oncologia e Hematologia Faculdade de Medicina de Ribeirão Preto USP Ribeirão Preto SP Brasil Departamento de Imagens Médicas, Oncologia e Hematologia - Divisão de Imagem, Faculdade de Medicina de Ribeirão Preto – USP , Ribeirão Preto , SP , Brasil

**Keywords:** Urinary Bladder Neoplasms, Diffusion Magnetic Resonance Imaging, Genitourinary Tract Anomalies [Supplementary Concept]

## Abstract

Bladder cancer (BCa) is one of the most common cancers worldwide and is also considered to be one of the most relapsing and aggressive neoplasms. About 30% of patients will present with muscle invasive disease, which is associated with a higher risk for metastatic disease. The aim of this article is to review the state of art imaging in Radiology, while providing a complete guide to urologists, with case examples, for the rationale of the development of the Vesical Imaging Reporting and Data System (VI-RADS), a scoring system emphasizing a standardized approach to multiparametric Magnetic Resonance Imaging (mpMRI) acquisition, interpretation, and reporting for BCa. Also, we examine relevant external validation studies and the consolidated literature of mpMRI for bladder cancer. In addition, this article discusses some of the potential clinical implications of this scoring system for disease management and follow-up.

## INTRODUCTION

Bladder cancer (BC) is the second common cancer within the genitourinary tract and the ninth most common malignancy in the World. It is even more prevalent within Western Europe ( 1 ). As of 2018, the number of global new cases and deaths from bladder cancers were estimated at approximately 550.000 and 200.000, respectively ( 2 ).

Smoking is the primary risk factor for bladder cancer and has been associated with over 55% of all cases in the United States ( 3 ). In addition, occupational exposure to polycyclic aromatic hydrocarbons and chlorinated hydrocarbons among paint and dye plant workers is the second most common risk factor ( 4 ). Also, the chronic irritation of the bladder mucosa, caused either by chronic urinary tract infections or stones is associated with an increased incidence of squamous cell cancers ( 5 ). With bladder cancer, there is a slight male predominance, ranging from 1.3/1.0 in Central Africa up to 4.0/1.0 in Southern Europe ( 1 , 5 ).

The staging of bladder cancer is of utmost importance. Usually, BC is staged as either non-muscle invasive BC (NMIBC) or muscle invasive BC (MIBC), based on the extension of the tumor invading the bladder wall. The proportion of MIBC, at initial diagnosis, is estimated between 25-30%. The invasion of the muscularis layer of the bladder has tremendous implications in the management and prognosis of the disease.

Despite all advances in the detection of small bladder lesions and carcinoma in situ (CIS), including narrow band imaging, fluorescence cystoscopy, and optical coherence, and the great interest about molecular assays, the chances of progression and recurrence in NMIBC patients are still very high and comparable to those seen at the end of 1990 ( 6 , 7 ). Some studies have showed that 10-20% of NMIBC patients at one time will eventually progress to MIBC, but roughly 50-70% will recur over time ( 8 ). Even when more aggressive management is chosen, the prognosis of BC is poor, with 5-year overall survive of 50% only, and with systemic (metastatic) disease up to 15% ( 1 , 9 ).

The relevance of BC, however, cannot be estimated only by those numbers. Not only is the mortality rate a significant concern in BC, but also the high rate of recurrence has a great impact on quality of life of a significant portion of patients with BC ( 8 ) and the high lifetime treatment-associated costs ( 10 , 11 ).

### The case for an Imaging Stratification Risk Score

Once the diagnosis of BC is made, the status of the bladder wall, according to major International Guidelines ( 12 - 14 ) is defined after tissue sampling performed at transurethral resection of bladder tumor (TURBT).

The need for appropriate staging tool could be scaled when Dutta et al. ( 15 ) demonstrated that for the patients who were under staged at initial diagnosis, the 5-year mortality rate was up to 30% higher compared to those correctly staged. One of the main limitations of TURBT for the diagnosis and staging of BC is its low sensitivity for assessment of MIBC ( 16 ). As showed, these clinically under staged patients are at higher risk for advance disease progression.

The clinical staging errors, at TURBT procedure, considering only T1 BC lesions, has been described and varies from 24 to 62%. In the series of Fristche et al. ( 17 ) and Ark et al. ( 18 ), the rate of incorrect staging at first TURBT was quite similar, 49.7% and 48.0%, respectively. In the study of Ark et al., multiple lesions and a history of prior TURBT were considered independent predictors of understaging at radical cystectomy (RC). Currently, en bloc TURBT has been proposed and advocated to reduce recurrence rates and the need for a second TURBT ( 19 ).

Besides these relevant implications for patient’s management, TURBT is a quite invasive procedure, although performed on an outpatient basis ( 12 ). The risk of bladder perforation is estimated by Herkommer et al. ( 20 ) in 1.1 to 5.3% patients. However, this could be underestimated, as demonstrated in a study by Balbay et al. ( 21 ), that found bladder perforations occurring in up to 58% of the cases, when using cystograms as the standard of reference.

Based on the lack of reliable molecular assays and the known limitations of TURBT as staging tool, there would be room for a staging technique that could accurately define the status of muscular layer, sparing patients from additional invasive procedures and, at the limit, potentially unnecessary surgery. For a long time, the use of imaging for local staging of BC has been limited to both Computed Tomography, and secondarily to Magnetic Resonance Imaging (MRI) ( 22 - 24 ).

However, based upon enhancement in soft tissue characterization with diffusion-weighted imaging (DWI) and dynamic-contrast enhancement (DCE), the accuracy of the multiparametric MRI for staging BC has significantly increased to greater than 90% ( 25 - 27 ).

### Standardization of imaging approach to BC - The VI-RADS initiative

In 2018, a multidisciplinary group of radiologists, urologists, pathologists and radiation oncologist, with an interest in bladder cancer, developed a scoring system ( 28 ) aimed to: 1) standardize the protocols for MR imaging of BC; 2) provide a structured reporting system to improve communication between referring physicians and radiologists, and; 3) provide a risk score for muscle layer invasion in BC. This initiative was named VI-RADS (Vesical Imaging-Reporting and Data System), which followed the precursors of “RADS”, the BI-RADS (Breast Imaging-Reporting and Data System) and the PI-RADS (Prostate Imaging-Reporting and Data System).

Briefly, the VI-RADS score system could be divided in three distinct, interconnected steps: patient preparation; exam acquisition protocol and images interpretation.

Patient preparation. Some important details are required in the preparation of patients undergoing a pelvic MRI for BC staging. These steps are all essentials to obtain the best results from the examination ( 28 ).

Antispasmodic agents are administered in order to minimize motion and inherent susceptibility artifacts ( 29 ). Patients are usually advised to void one to two hours before imaging and, depending on individual tolerance, to drink 0.5 to 1L of water before the examination. Indeed, adequate bladder distension is the major requirement. Ideally, the bladder should not be under or overdistended and a volume of 250 to 300mL is an ideal range for a good examination ( 30 ). Rapid sequences or real-time MRI can be used to monitor bladder distension. A good guide is to ensure proper visualization of the vesical dome on sagittal plane: an outward convex contour of the dome usually indicates an adequate distention. Without distention, the bladder wall can appear falsely thicker than usual, which, occasionally, could be misinterpreted as a lesion which can result in over staging ( 31 ).

### MRI protocol

The VI-RADS is largely based on multiparametric MRI, this multimodal approach was chosen to reduce the risk of error when staging a BC from one single sequence.

The evidence in literature suggests that high-field scanners, 1.5 and 3.0T, can be used indistinctively, as both generate high spatial resolution and signal-to-noise ratio ( 28 , 32 ). Here, the main recommendation is the use of a phased-array external, surface coil also with at least 16-channels.

The T2-weighted images were named structural category as these images, due to high contrast-resolution and excellent spatial resolution, are well suited for assessing the anatomy of the whole pelvis, including bladder and surrounding structures. These images can be acquired as 2D sequences in three planes (axial, coronal and sagittal) or can be acquired in a single volumetric ( 3 D) acquisition. The choice will be specific for every scanner, as the spatial and contrast resolution may show large variation depending on the vendor and generation of the scanner. The slice thickness should be kept thin, 3.0 to 4.0mm, maximum ( 33 ). T2WI is used to assess tumor detection, localization, evaluation of the size and morphology.

Diffusion-weighted images (DWI) is a functional technique based on the movements of water molecules in a given tissue or material ( 34 ). DWI has been used in virtually all abdominal examinations due to the significant provided information, particularly in oncological imaging, regardless of the site. It plays a critical role in prostate, liver and bladder cancer imaging ( 35 - 37 ). Although, at first glance, the T2 sequence provides better spatial resolution for assessing the depth of tumor extension, DWI has been proved more accurate for defining muscle layer status. Dynamic contrast enhanced (DCE) MRI is considered the dominant (defining) sequences, when disparities between sequences are found ( 25 , 38 ). A high B value (800-1000s/mm2) is usually required to visualize BC. Images are obtained in at least the axial plane; however, an additional plane (such as sagittal) is helpful for staging, especially for small lesions ( 39 ).

The DCE is the third key component of the VI-RADS and bladder MRI protocol ( 28 ). Its acquisition strongly relies on well-defined time points, which improves the differentiation between the inner layer (mucosa + lamina propria) from the muscularis propria (also referred to as detrusor muscle) ( 40 ). Although, anatomical evaluation is the major goal, it can be considered a functional technique, as it reflects vascularity and microvessel permeability and semi-quantitative analysis can be performed from this sequence. The best option is the use of an axial 3D T1-weigthed, gradient echo (GRE) sequence with fat-suppression that can be reformatted in other planes, due to high spatial resolution ( 41 ). Recently, a prospective study reported similar accuracy of a protocol without intravenous contrast media compared to multiparametric one, for the detection of muscle invasion ( 42 ). However, further studies are required for better evaluation of a biparametric approach for BC staging.

The original VI-RADS manuscript provides details of the technical requirements and the acquisition protocol of MRI, along with specific references ( 28 ).

### Interpretation and Reporting

The VI-RADS score has five categories ( 28 ), based upon degrees of invasion of the muscularis layer from highly unlikely, category 1, to very likely, category 5 ( Table-1 ).

**Table 1:** VI-RADS original description for SC-Structured Category (T2 images), Dynamic Contrast-Enhanced (DCE) and Diffusion-Weighted Imaging (DWI). Adapted from reference #27.

	Structured Category (T2)	DCE	DWI
1	Uninterrupted low SI line representing the integrity of muscularis propria (lesion <1.0 cm; e.g., exophytic tumor with or without stalk or thickened inner layer	No early enhancement of the muscularis propria (lesions corresponding to SC 1 findings)	Muscularis propria with intermediate continuous SI on DWI (lesion <1cm, hyperintense on DWI and hypointense on ADC, with or without stalk and/or low SI thickened inner layer on DWI)
2	Uninterrupted low SI line representing the integrity of muscularis propria (lesion >1cm; exophytic tumor with stalk and/or high SI thickened inner layer, when present, or sessile/broad-based tumor with high SI thickened inner layer, when present)	No early enhancement of muscularis propria with early enhancement of inner layer (lesions corresponding to SC 2 findings)	Muscularis propria with continuous intermediate SI on DWI (lesion >1cm, hyperintense on DWI and hypointense on ADC, with low SI stalk and/or low SI thickened inner layer on DWI, or broad-based/sessile tumor with low/intermediate SI thickened inner layer on DWI).
3	Lack of category 2 findings with associated presence of an exophytic tumor without stalk, or sessile/broad-based tumor without high SI thickened inner layer but with no clear disruption of low SI muscularis propria	Lack of category 2 findings (lesions corresponding to SC category 3 findings) but with no clear disruption of low SI muscularis propria.	Lack of category 2 findings (lesions corresponding to T2 category 3 findings) but with no clear disruption of low SI muscularis propria.
4	Interruption of low SI line suggesting extension of the intermediate SI tumor tissue to muscularis propria	Tumor early enhancement extends focally to muscularis propria	High SI tumor on DWI and low SI tumor on ADC extending focally to muscularis propria.
5	Extension of intermediate SI tumor to extravesical fat, representing the invasion of the entire bladder wall and extravesical tissues	Tumor early enhancement extends to the entire bladder wall and to extravesical fat	High SI tumor on DWI and low SI tumor on ADC extending to the entire bladder wall and extravesical fat.

The initial approach is usually done using T2-weigthed images, where the lesions present with intermediate signal, contrasting to the low signal of the muscle layer and the high signal of urine ( 43 ). This differentiation will be pursued in all sequences of a mpMRI of the bladder ( Figure- 1 ). An uninterrupted low signal will be the hallmark of categories 1 and 2, highly predictive of NMIBC, with category 2 reserved for lesions larger than 1.0cm ( Figure-2 ). On the other side, an unequivocally interrupted low signal is the typical finding indicating muscle invasion, reserved for categories 4 and 5 ( Figure-3 ). The latter is assigned when perivesical fat extension and involvement of adjacent structures are present. The category 3 is used when there is absence of category 2 findings, but when no obvious discontinuity of the muscle layer is observed.

**Figure 1:**
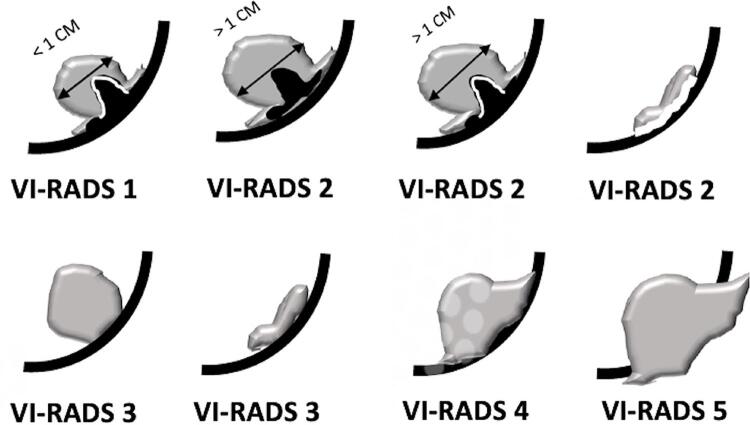
These pictures illustrate how structural categories (T2 images) of VI-RADS are assigned. The muscularis propria is presented as a thick black layer. The inner layer (urothelium + lamina propria) is a thin white layer. The tumors are shown in grey and the stalk, when present, in black, in continuity with the muscular layer. The inner layer is preserved in categories 1 and 2. In category 3, the inner layer is not seen, but there is no clear sign of muscle invasion. In categories 4 and 5, the tumors have extended to the muscular layer, and in VI-RADS 5, they go beyond, until perivesical fat.

**Figure 2:**
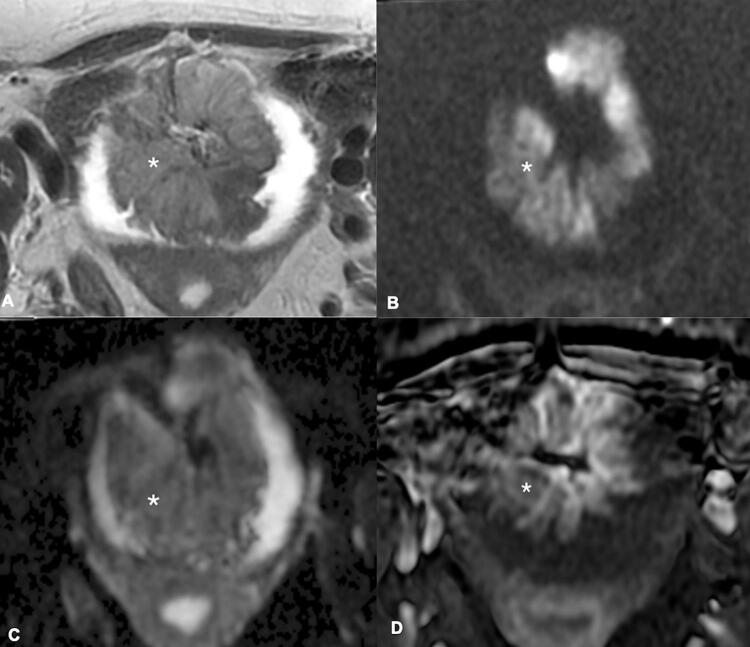
A 72-year-old woman presented with macroscopic hematuria. A) T2W image (axial plane) shows a large stalked mass at the anterior bladder wall. B and C) DWI (b=2000) and ADC maps show significantly restricted diffusion, not extending through the muscularis propria; the “inchworm” sign can be appreciated. D) DCE imaging shows early and heterogeneous enhancement of the lesions, not extending through the muscularis propria. The final VI-RADS score was 2. T2W, T2 weighted; DCE, dynamic contrast-enhanced; DWI, diffusion-weighted image; ADC, apparent diffusion coefficient.

**Figure 3:**
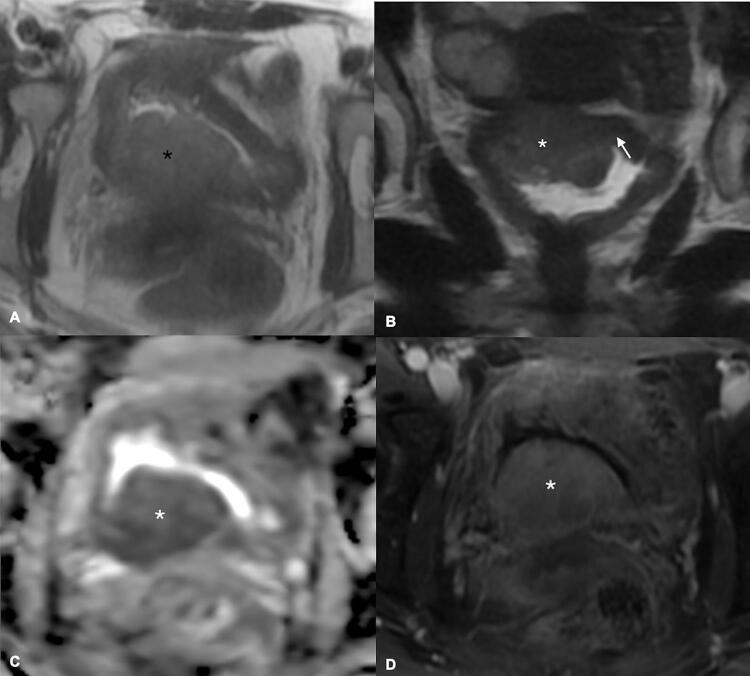
A 46-year-old, female, complains of frequency, urgency, and severe incontinence. A pelvic sonogram showed moderate to marked left hydronephrosis and asymmetric bladder wall thickening on the top portion of her bladder. A and B) Axial and Coronal T2-weighted MRI of the pelvis demonstrates a 4.4 x 3.6cm mass extending to muscle layer. C) ADC map in the axial plane, and D) T1 post-contrast, also in the axial plane, confirming that mass shows extension into the muscular layer. This was consistent with VI-RADS 4, confirmed after surgery.

**Figure 4:**
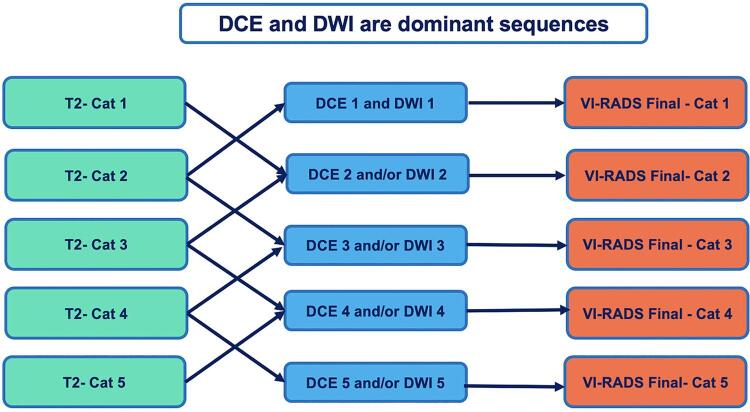
The decision algorithm for VI-RADS. When all categories are coincident, the final score is directly assigned. When classification in different sequences is discordant, DCE, and DWI are the dominant sequences and will prevail for the final classification. As seen in figure 4, DCE and DWI can upgrade or downgrade the initial classification found on T2 images.

The same approach is performed for DWI and DCE images. However, these two categories are the dominant sequences, therefore, in cases where there is discrepancy of findings between structural category (T2 images) and functional sequences (DWI and DCE), these two sequences will prevail, either for downgrading or upgrading the lesion ( 28 ). Accordingly, the final classification may be originated from several different combinations of T2, DWI and DCE categories as showed in Figure-4 .

The report of any vesical lesion should be done in a semi-structured model ( 44 ), following these steps: clinical indication; a brief description of the MRI protocol; findings description including lesion location, morphology, measurements, and signal characteristics, when scoring at T2, DWI and DCE is assigned. The evaluation for transmural extension, adjacent organ invasion (when present), and pelvic lymph nodes and bone status are also performed. Finally, the final category and comments should be provided to summarize the report.

### Validation Studies

The VI-RADS score system has been tested in several studies, from all over the World ( 45 - 56 ), either prospective or retrospective in nature. Two major points have been assessed in these initial studies: its reproducibility and its diagnostic accuracy for determining muscle layer invasion ( Table-2 ).

**Table 2:** Main validation studies published until March 2021.

Study/year		Country	Study type		Nature		#of patients	Interreader agreement			Sensitivity		Specificity		Accuracy		Standard of Reference
Ueno et al. 2019 ( 44 )*		Japan	Original Research		Retrospective		74	ICC=0.85			0.76 (Cat. ≥4) 0.88 (Cat. ≥3)		0.93 (Cat. ≥4) 0.77 (Cat. ≥3)		90		TURB
Barchetti et al. 2019 ( 45 )		Italy	Original Research		Retrospective		75	K=0.73			0.82 - 91 (Cat. ≥3)		0.85 - 0.89 (Cat. ≥3)		0.87 - 0.93		TURB
Wang et al. 2019 ( 46 )		China	Original Research		Retrospective		340	K=0.92			0.87 (Cat. ≥3)		0.97 (Cat. ≥4)		0.94		TURB, Cystectomy
Makboul et al 2019 ( 47 )		Egypt	Original Research		Prospective		50	K=0.87			0.78 (Cat. ≥3)		0.88 (Cat. ≥3)		0.83		TURB
Kim et al. 2019 ( 48 )		South Korea	Original Research		Retrospective		297	K=0.89 (T2) K=0.82 (DWI) K=0.85 (DCE)			0.91 (Cat. ≥4) 0.95 (Cat. ≥3)		0.76 (Cat. ≥4) 9.44 (Cat. ≥3)		N/A		TURB, Cystectomy
Del Giudice et al. 2019 ( 49 )		Italy	Original Research		Prospective		231	K=0.92			0.92 (Cat. ≥3)		0.91 (Cat. ≥3)		0.94		TURB, Cystectomy
Hong et al. 2020 ( 50 )		South Korea	Original Research		Retrospective		66	K=0.97			0.90 (Cat. ≥3)		1.0 (Cat. ≥3)		0.95		TURB, Cystectomy
Marchioni et al. 2020 ( 51 )		Italy	Original Research		Prospective		38	K=0.76			0.86 (Cat. ≥4)		0.87 (Cat. ≥4)		0.90		TURB
Liu et al. 2020 ( 52 )		China	Original Research		Retrospective		126	N/A			0.94 (Cat. ≥4)		0.92 (Cat. ≥4)		0.90		TURB, Cystectomy
Wang et al. 2020 ( 53 )		China	Original Research		Retrospective		220	N/A			0.92 (Cat. ≥4) 0.97 (Cat. ≥3)		0.95 (Cat. ≥4) 0.77 (Cat. ≥3)		0.96		TURB, Cystectomy
Sakamoto et al. 2020 ( 54 )		Japan	Original Research		Retrospective		176	K=0.43			0.63 (Cat. ≥4) 0.78 (Cat. ≥3)		0.96 (Cat. ≥4) 0.70 (Cat. ≥3)		0.86		TURB
Metwally et al. 2021 ( 55 )		Egypt	Original Research		Prospective		331	K=0.93			0.84 (Cat. ≥4)		0.90 (Cat. ≥4)		0.94		TURB
Woo et al. 2020 ( 57 )		USA	Meta-analysis				1770 (6 studies)	K=0.81 - 0.92 ICC=0.85			0.83		0.90		0.94		TURB, Cystectomy
Luo et al. 2020 ( 58 )		China	Meta-analysis				1064 (6 studies)	N/A			0.90		0.86		0.93		TURB, Cystectomy

*number of citation in the text.

N/A = not available.

TURB = Transurethral resection of bladder.

The interobserver agreement for VI-RADS can be considered a major strength for the system. It has been reported in the range of optimal to almost perfect, varying from 0.73 up to 0.92, regardless of the experience of the readers. In a recent meta-analysis of Del Giudice et al. ( 57 ), focusing on the reproducibility of VI-RADS, the pooled weighted mean kappa score (κ) was 0.83 (95% Confidence Interval: 0.78-0.88), in spite of a significant heterogeneity in the studies included in the systematic review. Of importance here is to remember that reproducibility, in a broad sense, may encompass the variations across different scanners and centers, with different levels of experience, which may influence the adoption of a new classification system.

The diagnostic accuracy of VI-RADS has been evaluated in two recent meta-analysis. In the study of Woo et al. ( 58 ), six studies, two prospective, were included and the pooled sensitivity was 0.83 (95% confidence interval, 0.70-0.90) and pooled specificity was 0.90 (0.83-0.95), and the accuracy, measured by the area under the ROC curve, was 0.94 (0.91-0.95). Luo et al. ( 59 ) also included six studies (five were the same as in the study of Woo et al.), including the same two prospective studies, and pooled sensitivity, specificity, and diagnostic accuracy (again by AUC) were, respectively, 0.90 (0.86-0.94), 0.86 (0.71-0.94), and 0.93 (0.91-0.95) using VI-RADS 3 as the cutoff value for muscle invasion and, 0.77 (0.65-0.86), 0.97 (0.88-0.99), and 0.92 (0.89-0.94) when VI-RADS 4 was the cut off for invasion. In both meta-analysis, there was a significant study heterogeneity. Woo et al. ( 58 ) indicated the number of patients in the study, the magnetic field strength of the scanners (3.0 vs. 1.5T), image slice thickness (3 vs. 4mm) in T2 images, and VI-RADS cutoff score, from 3 or 4 as the major source of heterogeneity. In the study of Luo et al. ( 59 ), study design (retrospective or prospective) and surgical pattern of standard of reference were the main source of the heterogeneity.

The definition of which score should assumed as indicative of invasion of muscle layer varies, as different scores can be chosen according to different clinical scenarios. For instance, VI-RADS 3 could be used as the cutoff value when dealing with patients with high pre-test probability of muscle invasion, including, but not limited to, patients with high-grade, recurrent, multiple and or larger lesions (>3.0cm). On the other side, VI-RADS 4 could be defined as the cut off, in clinical settings requiring higher specificity, e.g., more aggressive treatment options are being considered.

Both studies showed similar results of a previous meta-analysis, carried out in 2017, before VI-RADS release, including 24 studies, showing pooled sensitivity of 0.92 (95% CI 0.88-0.95) and specificity of 0.87 (95% CI 0.78-0.93). Here, the appeal for the use of VI-RADS relies on the future gains of using a standardized approach for image acquisition and for reporting BC lesions assessed by mpMRI. The potential gain in the reproducibility, as the performance of less skilled readers tend to increase when an established system is used, as demonstrated by the comparison of PI-RADS and Likert scale ( 60 ).

Of note, Del Giudice et al. ( 61 ) investigated the role of VI-RADS score 5 in predicting time-to-cystectomy (TTC) outcomes. Authors showed, not only that VI-RADS is valid and reliable in differentiating patients with extravesical disease, but also that the identification of a VI-RADS score of 5 implies in a significant delay in TTC, independently from other clinicopathological features.

VI-RADS also provided possible alternatives and decision aids in the treatment of BCa during the COVID-19 emergency setting, to minimize potential exposure to the infection by avoiding hospital admissions: patients with NMIBC and preoperative VI-RADS score of 1-2 were directed to appropriate adjuvant intravesical therapy for follow-up, rather than a secondary resection of the tumor, considering the low risk of understaging ( 62 ).

Recently, the first multi-institutional, multi-reader study, authored by Ueno et al. ( 63 ), who observed moderate to substantial interobserver agreement and a pooled AUC of 0.87 among radiologists of different levels of expertise using VI-RADS, again confirming the existing high reproducibility of score in the “real life” clinical practice (different scans and different reader’s experience).

### Perspectives for the use of mpMRI and VI-RADS in Bladder Cancer

The original suitability of VI-RADS system was limited to patients not previously surgically manipulated, to avoid post-procedures changes influencing the final classification ( 28 ). This requirement limits the applicability of the score system, as frequently, patients have already submitted to TURBT. Considering the relevance of expanding the use of VI-RADS, new data on this topic is expected to be coming in the near future, with emphasis on the accuracy of MRI and VI-RADS scoring in differentiating inflammatory changes secondary to the surgical procedure from malignant findings ( 64 - 67 ).

A second issue for potential VI-RADS updating is the incorporation of associated findings. Currently, there is no place for citing these features, some of them with a potential to change management of the lesion, for instance, hydronephrosis ( 68 ).

Another potential use of mpMRI and VI-RADS is to stratify patients diagnosed with high-risk NMIBC at first TURBT ( 69 , 70 ). The risk of muscle layer invasion at radical cystectomy, in these patients is estimated in about 30% ( 71 , 72 ). In this setting, the use of VI-RADS for risk stratification and discrimination of those who should undergo secondary tumor resection and those who can be spared might be assessed in the near future. A trial assessing the value of mpMRI in this clinical setting was initiated in the United Kingdom ( 73 ), where the “Bladder-Path” study was designed to divide patients with confirmed BC after first TURBT, into a group with probable NMIBC, receiving current standard approach, from another group composed of patients with risk factors for MIBC, who will proceed to mpMR imaging for differentiation between MIBC and NMIBC.

With regards to the high rate of recurrence for BC, the post-treatment surveillance is another potential use of mpMRI ( 72 ). Although, cystoscopy is the gold-standard in the follow-up of these patients, a non-invasive tool could be helpful, especially when a local recurrence is suspected. In follow-up period, inflammatory changes after a TURBT may persist for up to 24 months ( 66 ) and could be misinterpreted, mostly within 2 weeks from the procedure, as residual or recurrent disease especially on T2-weighted images. Nonetheless, DCE and especially DWI are crucial for the correct interpretation.

The medical treatment for NMIBC and MIBC includes chemotherapy, immune checkpoint inhibitors and Bacillus Calmette-Guerin (BCG) intravesical instillations ( 74 ). However, considering the limitations of applying solid tumors response criteria in the bladder to evaluate tumor burden before and after medical treatment, mpMRI has been useful in the assessment of these patients as demonstrated by a marked increase in the ADC values with complete response after neoadjuvant chemotherapy ( 75 - 77 ). Also, considering the response to immunotherapy, Necchi et al. ( 78 ) demonstrated the promising role of MRI in the evaluation of response to therapy before and after immunotherapy. However, it did so apply a dichotomic method, which implied fewer promising outcomes from the combined complete/partial responder’s assessment (i.e., pT≤1). Instead, the use of a five-scale assessment score for response to system therapy might provide a model to define the complete spectrum of pathological treatment response among MIBC patients ultimately undergoing RC.

## CONCLUSIONS

The technological innovation of MR imaging has advanced the assessment of bladder cancer. These ongoing developments have yet to be better defined but arguably have the potential to change how BC is staged and monitored. In the future, MR findings can be incorporated to increase the accuracy of the traditional prediction models as the EORTC, CUETO, and EAU risk stratification. The use and implication of VI-RADS will improve the communication in the diagnosis, staging and surveillance of patients with bladder cancer.
